# Genetic alterations in hepatocellular carcinomas: association between loss of chromosome 4q and *p53* gene mutations

**DOI:** 10.1038/sj.bjc.6690321

**Published:** 1999-04

**Authors:** A Rashid, J-S Wang, G-S Qian, B-X Lu, S R Hamilton, J D Groopman

**Affiliations:** Division of Gastrointestinal/Liver Pathology, Department of Pathology, Ross Building, Room 632; The Johns Hopkins Oncology Center, 720 Rutland Avenue, Baltimore, MD 21205-2196, USA; Department of Environmental Health Sciences, The Johns Hopkins University, School of Hygiene and Public Health; Shanghai Cancer Institute, Shanghai andQidong Liver Cancer Institute, Qidong, People's Republic of China

**Keywords:** p53 gene, loss of heterozygosity, hepatitis B virus, aflatoxin, hepatocellular carcinomas

## Abstract

The major risk factors for hepatocellular carcinomas (HCC) in high incidence areas include infection with hepatitis B and C viruses (HBV, HCV) and exposure to aflatoxin. Genetic alterations in 24 liver resection specimens from Shanghai and Qidong were studied. Hepatitis B virus was integrated in all patient samples, and a null phenotype for the GSTM1 enzyme was present in 63% of patients. Alteration of p53 was present in 95% (23/24) of cases: mutations of the p53 gene in 12 HCC, p53 overexpression in 13 and loss of heterozygosity (LOH) of chromosome 17p in 17. All seven HCCs with a p53 mutation from Qidong and three of five from Shanghai had the aflatoxin-associated point mutation with a G to T transversion at codon 249, position 3. No HCC had microsatellite instability. LOH of chromosome 4q, 1p, 16q and 13q was present in 50%, 46%, 42% and 38%, respectively, and 4q was preferentially lost in HCCs containing a p53 mutation: LOH of 4q was present in 75% (9/12) of HCC with, but only 25% (3/12) of HCC without, a p53 gene mutation (*P* = 0.01). These data indicate a possible interaction between p53 gene mutation and 4q loss in the pathogenesis of HCC. © 1999 Cancer Research Campaign


*British Journal of Cancer* (1999) **80**(1/2), 59–66
© 1999 Cancer Research Campaign
Article no. bjoc.1998.0321

### Genetic alterations in hepatocellular carcinomas: association between loss of chromosome 4q and *p53* gene mutations

**A Rashid[1,2], J-S Wang[3], G-S Qian[4], B-X Lu[5], SR Hamilton[1,2] and JD Groopman[2,3]**

[1]Division of Gastrointestinal/Liver Pathology, Department of Pathology, Ross Building, Room 632; [2]The Johns Hopkins Oncology Center, The John Hopkins School of Medicine, 720 Rutland Avenue, Baltimore, MD 21205-2196, USA; [3]Department of Environmental Health Sciences, The Johns Hopkins University, School of Hygiene and Public Health; [4]Shanghai Cancer Institute, Shanghai, and [5]Qidong Liver Cancer Institute, Qidong, People's Republic of China

**Summary** The major risk factors for hepatocellular carcinomas (HCC) in high incidence areas include infection with hepatitis B and C viruses (HBV, HCV) and exposure to aflatoxin. Genetic alterations in 24 liver resection specimens from Shanghai and Qidong were studied. Hepatitis B virus was integrated in all patient samples, and a null phenotype for the GSTM1 enzyme was present in 63% of patients. Alteration of p53 was present in 95% (23/24) of cases: mutations of the p53 gene in 12 HCC, p53 overexpression in 13 and loss of heterozygosity (LOH) of chromosome 17p in 17. All seven HCCs with a p53 mutation from Qidong and three of five from Shanghai had the aflatoxin-associated point mutation with a G to T transversion at codon 249, position 3. No HCC had microsatellite instability. LOH of chromosome 4q, 1p, 16q and 13q was present in 50%, 46%, 42% and 38%, respectively, and 4q was preferentially lost in HCCs containing a p53 mutation: LOH of 4q was present in 75% (9/12) of HCC with, but only 25% (3/12) of HCC without, a p53 gene mutation ($P = 0.01$). These data indicate a possible interaction between p53 gene mutation and 4q loss in the pathogenesis of HCC.

**Keywords:** p53 gene; loss of heterozygosity; hepatitis B virus; aflatoxin; hepatocellular carcinomas

Hepatocellular carcinoma (HCC) is one of the most common causes of cancer death worldwide (Yu and Chen, 1994). The incidence of HCC shows considerable geographical variation, and there is a very high incidence of HCC in China, sub-Saharan Africa and Southeast Asia but a low incidence in North America and Europe. Epidemiological studies in high-risk populations have identified chronic hepatitis B virus (HBV) and chronic hepatitis C virus (HCV) infections as well as dietary exposure to aflatoxin $B_1$ ($AFB_1$) as major factors in the aetiology of this disease (Groopman et al, 1996). Glutathione S-transferase M1 (GSTM1) and microsomal epoxide hydrolase are implicated in detoxification of $AFB_1$, and increased frequency of mutant alleles is present in patients with HCC (McGlynn et al, 1995). In the People's Republic of China, Qidong has high exposure to aflatoxin while Shanghai has intermediate exposure (Qian et al, 1994).

Mutations of the p53 tumour suppressor gene are common in HCC, and a distinct pattern of mutations in this gene has been described. HCC from China and sub-Saharan Africa, areas with a high incidence of chronic HBV infection and dietary exposure to $AFB_1$, have a G–T transversion at codon 249 of the p53 gene that is present in 10–50% of tumours (Bressac et al, 1991; Hsu et al, 1991; Ozturk et al, 1991; Coursaget et al, 1993). The biology of this mutant p53 gene product has been characterized in a transgenic mouse model (Ueda et al, 1995), in murine and human hepatocyte cell lines (Mace et al, 1991; Dumenco et al, 1995), and in human hepatoma cell lines (Aguilar et al, 1993; Ponchel et al, 1994). In HCC from areas with low dietary exposure to $AFB_1$, p53 mutations are seen at a much lower frequency, and a broader spectrum of mutations which do not affect a particular codon is evident (Nose et al, 1993; Hayashi et al, 1995; Kubicka et al, 1995; Shi et al, 1995). p53 overexpression due to prolonged half-life of many mutated p53 gene products and loss of heterozygosity (LOH) of 17p are also present in HCCs with a p53 mutation (Nose et al, 1993; Hsu et al, 1994; Bourdon et al, 1995; Yumoto et al, 1995). In HCCs developing in a person with chronic HBV infection, hepatitis B x antigen (HBxAg), a HBV-encoded protein, may bind p53 protein and cause functional inactivation of p53 (Greenblatt et al, 1997). Thus, gene–chemical, gene–virus and chemical–virus interactions may have great significance in the early onset and rapid lethality of HCC.

Many chromosomal aberrations are reported frequently in HCCs, including LOH of chromosome 1p, 4p, 4q, 6q, 8p, 9p, 11p, 13q, 16p, 17p and 22q by cytogenetics, restriction length fragment polymorphism (RFLP) analysis and microsatellite analysis (Wang et al, 1988; Buetow et al, 1989; Tsuda et al, 1990; Zhang et al, 1990, 1994; Fujimori et al, 1991; Simon et al, 1991; Walker et al, 1991; Emi et al, 1992; Slagle et al, 1993; Takahashi et al, 1993; Yeh et al, 1994, 1996; De Suza et al, 1995; Nasarek et al, 1995; Kuroki et al, 1995*a*, 1995*b*; Yumoto et al, 1995; Chen et al, 1996; Leon et al, 1996; Becker et al, 1996; Boige et al, 1997; Marchio et al, 1997; Nagai et al, 1997). Previous studies have shown an association between p53 gene mutations, LOH, metabolic enzyme polymorphisms, or hepatitis viruses in HCCs from Shanghai and Qidong, the People's Republic of China, representing areas of intermediate and high exposure to aflatoxin, respectively. The purpose of this study was to explore interactions among multiple aetiological and genetic variables.

*Received 19 June 1998*
*Revised 14 October 1998*
*Accepted 4 November 1998*

*Correspondence to:* A Rashid

### MATERIALS AND METHODS

### Case material

Fresh tissue was collected from patients diagnosed with HCC undergoing hepatic resection in Shanghai (*n* = 10) and Qidong (*n* = 14), People's Republic of China (Table 1). Neoplastic and non-neoplastic liver tissue were frozen in liquid nitrogen and kept at –70°C until processing.

### Histopathological examination

Representative sections of neoplastic and non-neoplastic frozen tissues were thawed, fixed in formalin and embedded in paraffin. Standard haematoxylin and eosin and Masson's trichrome sections were reviewed by one of us (AR). Each non-neoplastic liver tissue was scored for chronic hepatitis (defined by lobular and portal chronic inflammation, hepatocyte degeneration and fibrosis) and cirrhosis. Each HCC was scored for tumour differentiation.

### Immunohistochemistry for hepatitis B surface and core antigens and hepatitis C virus antigen

Immunohistochemistry was performed on thawed, formalin-fixed, paraffin-embedded tissue using a standard avidin–biotin complex (ABC) immunohistochemical technique after antigen retrieval by a heat-induced epitope retrieval method (Bankfalvi et al, 1994). The primary antibodies used in the present study were hepatitis B surface antigen (HBsAg; Zymed Industries Inc., San Fransisco, CA, USA) at 1:1000 dilution, hepatitis B core antigen (HBcAg; DAKO Corporation, Carpinteria, CA, USA) at 1:10 000 dilution, HCV (Signet Laboratories, Dedham, MA, USA) at 1:100 dilution and p53 (D07, DAKO Corporation, Carpinteria, CA, USA) at 1:100 dilution. Immunohistochemistry was performed on 5-μm sections on Probe-on Plus slides (Fisher Scientific), which were mounted and baked for 20 min at 60°C. Sections were reacted with primary antibody for 2 h. As a negative control, adjacent sections were treated with isotype-matched antibody using equivalent conditions. Secondary reagents were obtained from the Vector Elite ABC kit (Vector, Burlingham, CA, USA) and diaminobenzidine was used as chromogen with light haematoxylin counterstain.

### DNA preparation

Genomic DNA was extracted from microdissected neoplastic and non-neoplastic frozen tissue using a commercial kit according to the manufacturer's instructions (QIAamp tissue kit, Qiagen Inc., Valencia, CA, USA).

### PCR amplification for HBsAg, HBxAg and GSTM1 genes

Integration of HBV was determined by PCR amplifications of HBsAg and HBxAg gene segments as described (Hsu et al, 1993) from genomic DNA prepared from the neoplastic and non-neoplastic tissue. GSTM1 was genotyped by two separate polymerase chain reaction (PCR) amplifications of genomic DNA from non-neoplastic liver tissue to amplify a 273 base pair segment of exons 4 and 5 (Comstock et al, 1990) and a 650 base pair segment of exons 3–5 using oligonucleotides as described previously (Brockmoller et al, 1993). The PCR products were analysed on a 1.5% agarose gel. For each assay, a band represented the presence of DNA encoding a viral protein or an active enzyme, whereas absence of a band indicated absence of the DNA segment encoding a viral protein or the presence of a disabling deletion mutation of enzyme. Null genotype, represented by deletion of the GSTM1 locus, was discriminated from the presence of GSTM1 in the hemizygous or homozygous state.

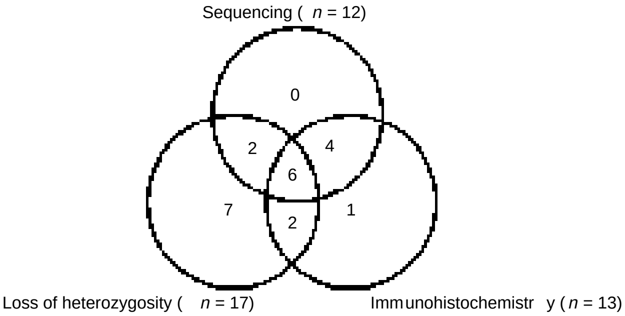

**Figure 1** Abnormalities of p53 gene in hepatocellular carcinomas. Mutation of the p53 gene by DNA sequencing, loss of heterozygosity (LOH) of microsatellite markers on the short arm of chromosome 17 and overexpression of p53 by immunohistochemistry

### Sequencing of epoxide hydrolase and p53 genes

A segment of epoxide hydrolase exon 3 was amplified by a PCR reaction of non-neoplastic genomic DNA using the oligonucleotides 5′-CCC CAC CTT TGG AGG ACA GC-3′, and 5′-CCC TTC AAT CTT AGT CTT G-3′. The PCR product was sequenced using the oligonucleotide 5′-GTC ATC TCC TAC TGG GGG-3′. Exons 2–9 of p53 were sequenced as described previously (Redston et al, 1994). p53 mutations were confirmed by a repeat PCR amplification and sequencing, and non-neoplastic DNA was sequenced to rule out a germline p53 mutation.

### Microsatellite markers and LOH

Microsatellite markers were obtained from Research Genetics (Huntsville, AL, USA). LOH was analysed by PCR amplification of dinucleotide repeats or tandem repeats (Table 2) present on chromosomes 1p, 4q, 13q, 16q and 17p. A PCR-based microsatellite-repeat assay was carried out in 96-well plates for 38 cycles per minute using PCR Master (Boehringer Mannheim, GamH, Germany), as described previously (Rashid and Hamilton, 1997). Loss of a chromosomal marker was considered to be present when the PCR assay showed absence or more than 50% loss of intensity of a heterozygous band from a tumour sample as compared with the corresponding non-tumour sample. Complete or partial loss of the chromosomal arm was determined from the pattern of markers with LOH. Fractional allelic loss (FAL) was calculated for each tumour as percentage of microsatellite markers lost among informative markers.

### Statistical analysis

Statistical significance was calculated by Fisher's exact test using True Epistat (Epistat Services, Richardson, TX, USA). All *P*-values reported are two-sided.

© Cancer Research Campaign 1999

**Table 1** Histopathological features, hepatitis B virus status, alterations of p53 gene, and polymorphism of aflatoxin enzymes in HCC

| Tumor no. | Cirrhosis | Differentiation of HCC | PCR HBsAg/ HbxAg | HBsAg IHC[a] | HBcAg IHC | p53 IHC | p53 sequencing amino acid (nucleotides) | GSTM1 null phenotype[b] | Epoxide hydrolase[c] |
|---|---|---|---|---|---|---|---|---|---|
| *Shanghai* | | | | | | | | | |
| 1C-221 | – | Moderate | + | N+ | – | + | 174 Arg-Trp (AGG-TGG) | – | H/H |
| 1C-223 | – | Moderate | + | – | – | – | Wild-type | – | Y/Y |
| 1C-224 | – | Moderate | + | T/N + | – | + | 249 Arg-Ser (AGG-AGT) | – | H/H |
| ID-098 | + | Poor | + | T/N + | – | – | Wild-type | + | Y/H |
| ID-099 | + | Poor | + | T/N + | – | – | Wild-type | + | H/H |
| ID-100 | + | Moderate | + | T/N + | – | + | 249 Arg-Ser (AGG-AGT) | – | Y/H |
| ID-106 | + | Moderate | + | – | – | – | Wild type | + | H/H |
| ID-107 | + | Poor | + | N+ | – | – | Wild type | + | Y/Y |
| ID-109 | – | Poor | + | N + | – | + | 249 Arg-Ser (AGG-AGT) | – | Y/H |
| ID-121 | + | Moderate | + | N + | + | – | 266 Gly-Arg (GGA-CGA) | + | Y/Y |
| Total (*n* = 10) | 6 | | 10 | 8 | 1 | 4 | 5 | 5 | 4H/H, 4 Y/H |
| *Qidong* | | | | | | | | | |
| 94–18 | + | Poor | + | N + | – | + | Wild type | + | Y/Y |
| 94–20 | + | Moderate | + | T/N + | – | + | 249 Arg-Ser (AGG-AGT) | + | Y/Y |
| 94–21 | – | Moderate | + | – | – | + | Wild type | + | H/H |
| 94–24 | – | Poor | + | N + | – | + | 249 Arg-Ser (AGG-AGT) | – | Y/H |
| 94–25 | + | Moderate | + | N + | – | – | 249 Arg-Ser (AGG-AGT) | – | Y/Y |
| 94–28 | + | Poor | + | N + | – | + | 249 Arg-Ser (AGG-AGT) | + | Y/Y |
| 95–2 | – | Poor | + | – | – | + | 249 Arg-Ser (AGG-AGT) | – | Y/H |
| 95–3 | + | Poor | + | N + | + | – | Wild type | + | Y/Y |
| 95–4 | + | Poor | + | – | – | + | 249 Arg-Ser (AGG-AGT) | + | H/H |
| 95–6 | + | Moderate | + | N + | – | – | Wild type | + | Y/Y |
| 95–7 | + | Moderate | + | N + | – | + | Wild type | + | Y/H |
| 95–8 | + | Moderate | + | T/N + | – | + | 249 Arg-Ser (AGG-AGT) | + | Y/Y |
| 95–9 | + | Moderate | + | N + | – | – | Wild type | – | H/H |
| 95–12 | + | Moderate | + | T/N + | – | – | Wild type | + | Y/Y |
| Total (*n* = 14) | 11 | | 14 | 11 | 1 | 9 | 7 | 10 | 3 H/H, 3 Y/H |

[a]T = tumour; N = non-tumour. [b]Null phenotype denotes deletion of exons 4–5. [c]H = amino acid histidine at position 114 (a polymorphism with a less active enzyme), Y = tyrosine (wild-type enzyme).

### RESULTS

### Histopathological assessment

Fifty-eight per cent (14/24) of HCCs were moderately differentiated and 42% (10/24) were poorly differentiated (Table 1). Cirrhosis was present in non-neoplastic liver in 71% (17/24) of patients, and chronic hepatitis was present in all cases.

### Hepatitis B and C virus status

DNA segments encoding HBsAg and HBxAg could be amplified from the neoplastic and non-neoplastic DNA from all 24 cases of HCCs, indicating integration of viral DNA (Table 1). Immunohistochemistry for HBsAg was positive in non-neoplastic liver in 79% (17/24) of cases, and in 37% (7/24) of HCCs (Table 1). Immunohistochemistry for HBcAg was positive in only two non-neoplastic liver samples, and immunohistochemistry for hepatitis C virus antigen was negative in all neoplastic and non-neoplastic samples.

### GSTM1 and epoxide hydrolase genotype

Null genotype for GSTM1 was present in 63% (15/24) of patients (Table 1) versus 41% in a control population in Shanghai in a previous study (McGlynn et al, 1995). The remaining nine (37%) patients had at least one copy of the functional enzyme.

Fifty-four per cent (13/24) of patients had an abnormal genotype of epoxide hydrolase (Table 1). The wild-type sequence with homozygous tyrosine at codon 113 was replaced by a homozygous histidine, representing a polymorphic phenotype with less active enzyme, in 29% (7/24) of patients, and a heterozygous histidine/tyrosine was present in 25% (6/24, Table 1). These frequencies were not statistically different from a control population in Shanghai: in a previous study (McGlynn et al, 1995), a homozygous histidine at codon 113 was present in 26% of control population in Shanghai, a heterozygous histidine/tyrosine in 40%, and a homozygous tyrosine wild-type in 34% respectively.

Six patients had mutant alleles in both GSTM1 and epoxide hydrolase. Ninety-two per cent (22/24) of patients had a null GSTM1 genotype and/or had a histidine in epoxide hydrolase gene.

### Mutations of p53 gene, overexpression of p53 gene product by immunohistochemistry and LOH of chromosome 17p

Abnormality of p53 was the most common alteration in this set of HCCs, present in 95% (23/24) of cases as determined by genomic sequencing of exons 2–9, overexpression of p53 by immunohistochemistry, or LOH of chromosome 17 p (Figure 1). Fifty per cent (12/24) of HCCs had mutations in exons 2–9 of the p53 gene (Table 1). All seven HCCs with a p53 mutation from Qidong and three of five HCCs from Shanghai had point mutations with a G to

*© Cancer Research Campaign 1999* 

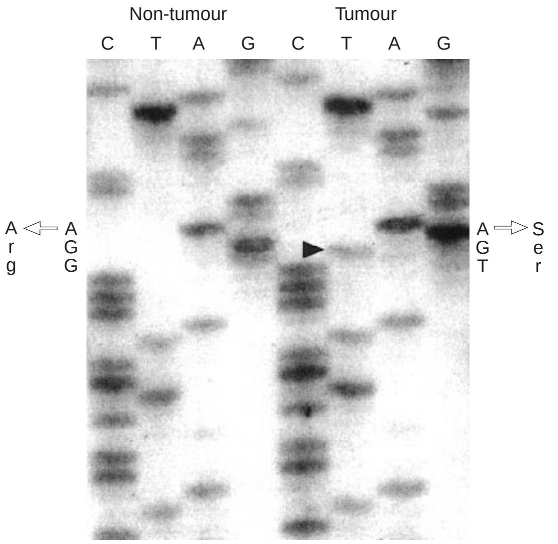

**Figure 2** Sequence of p53 gene, exon 7, showing a G to T transversion at codon 249 (substitution of amino acid arginine with serine) in a hepatocellular carcinoma. This mutation is frequent in hepatocellular carcinomas from high aflatoxin exposure areas

T transversion at codon 249, position 3 (substitution of amino acid arginine with serine, Figure 2), indicative of aflatoxin exposure. The remaining two HCCs from Shanghai had point mutations with a A to T transversion at codon 174, position 1 (substitution of glycine with arginine) and a G to C transversion at codon 266, position 1 (substitution of arginine with tryptophan). No germline p53 mutations were present.

Fifty-four per cent (13/24) of HCCs showed nuclear staining by p53 immunohistochemistry (Table 1), including 83% (10/12) of HCCs with a p53 mutation and 25% (3/12) with no mutation identified in exons 2–9 of the p53 gene ($P$ = 0.01, odds ratio (OR) 15, 95% confidence interval (CI) 1.5 and 190).

LOH of chromosome 17 p was present in 70% (17/24) of HCCs. 17p LOH was present in 67% (8/12) of HCCs with a p53 mutation, 75% (9/12) without an identified p53 mutation, 67% (8/12) with positive p53 immunohistochemistry and 75% (9/12) with negative p53 immunohistochemistry (Figure 2). Twenty-nine per cent (7/24) of HCCs showed 17p LOH without either p53 mutation or positive immunohistochemistry, but no case with an identified p53 mutation lacked both p53 overexpression and 17p loss.

### Microsatellite instability and LOH of chromosomes 1p, 4q, 13q and 16q

DNA replication errors (RER, microsatellite instability), indicative of alteration of mismatch repair genes, was not present in these HCCs. LOH of chromosome 1p was present in 46% (11/24) of patients (Table 2, Figure 3). The telomeric markers D1S160, D1S170 and D1S186 showed allelic loss in 44%, 40% and 50% of patients with informative loci, respectively, but the centromeric marker AMY2B did not show any allelic loss. LOH of chromosome 4q was present in 50% (12/24) of HCCs with informative loci (Figure 4). Eighty-three per cent (10/12) of these HCCs showed loss of two or more markers indicating a broad area of 4q deletion and five of these showed retention of heterozygosity in an intervening marker. LOH of chromosome 13q between the loci 13q12.3 and 13q21.1 was present in 38% (9/24) of HCCs, and LOH of chromosome 16q between 16q12.1 and 16q24.2 was present in 42% (10/24) of HCCs. Fractional allelic loss (FAL) ranged from 0% to 91% with a median of 32%.

LOH of chromosome 4q was present in 75% (9/12) of HCCs with a p53 gene mutation, but in only 25% (3/12) of HCCs without a p53 gene mutation (Figure 3; $P$ = 0.01, OR 9, 95% CI 1.1 and 84.6). Seven of the HCCs with 4q loss had the aflatoxin-associated G–T transversion at codon 249, position 3. No association of aflatoxin-modifying enzymes with loss of chromosomal arms or concordant losses of chromosomal arms were identified.

| | | IC221 | ID100 | ID109 | ID121 | 94-20 | 94-24 | 95-2 | 95-4 | 95-8 | IC2 24 | 94-25 | 94-28 | IC2 23 | 95-3 | 95-9 | ID 098 | ID 099 | ID 106 | ID 107 | 94-18 | 94-21 | 95-6 | 95-7 | 95-12 |
|---|---|---|---|---|---|---|---|---|---|---|---|---|---|---|---|---|---|---|---|---|---|---|---|---|---|
| **p53 mutation** | | + | + | + | + | + | + | + | + | + | + | + | + | - | - | - | - | - | - | - | - | - | - | - | - |
| **4q loss** | | + | + | + | + | + | + | + | + | + | - | - | - | + | + | + | - | - | - | - | - | - | - | - | - |
| D1S160 | 1p36.2 | ○ | NI | NI | ○ | ○ | NI | ○ | ● | NI | NI | NI | NI | NI | NI | ● | ● | NI | ● | NI | ○ | NI | NI | NI | NI |
| D1S170 | 1p36.2 | ○ | ○ | ● | NI | ○ | NI | ○ | ● | NI | ○ | ○ | NI | ● | ○ | NI | ○ | ● | ○ | ○ | NI | ○ | NI | NI | NI |
| D1S186 | 1p | ● | ○ | NI | ○ | ○ | NI | ○ | ○ | ● | ○ | ● | ● | ● | ○ | NI | ● | ○ | NI | ○ | NI | ○ | ○ | ○ | ○ |
| AMY2B | 1p21 | NI | ○ | ○ | ○ | NI | ○ | NI | ○ | NI | NI | NI | ○ | ○ | NI | NI | ○ | NI | ○ | ○ | ○ | ○ | ○ | ○ | NI |
| D4S395 | 4q12-13 | ● | NI | NI | ● | NI | NI | ● | NI | ● | ○ | ○ | ○ | ● | NI | ● | ○ | ○ | ○ | ○ | ○ | ○ | NI | ○ | ○ |
| D4S411 | 4q23-25 | ● | ● | NI | ○ | ● | ● | ● | ○ | NI | NI | ○ | ○ | ○ | ● | ● | ○ | NI | NI | ○ | NI | NI | NI | ○ | NI |
| D4S427 | 4q26-28 | ● | NI | ● | ● | ● | ● | ● | ○ | ○ | NI | ○ | ○ | ● | ● | NI | NI | NI | ○ | NI | NI | NI | ○ | NI | ○ |
| D4S422 | 4q28 | ● | ○ | ○ | NI | ● | ○ | NI | ● | ○ | ○ | ○ | ○ | ● | ○ | NI | NI | NI | NI | ○ | NI | NI | ○ | ○ | NI |
| D4S415 | 4q32 | ● | ○ | ● | ● | ● | NI | ● | ● | NI | ○ | NI | NI | NI | ● | ● | NI | ○ | ○ | NI | ○ | NI | NI | NI | ○ |
| D13S260 | 13q12.3 | NI | ○ | ○ | ○ | NI | ● | ○ | ○ | NI | NI | ○ | ○ | ○ | ○ | NI | ○ | ● | ○ | NI | ○ | ○ | ○ | NI | NI |
| D13S126 | 13q14.1-14.3 | ○ | NI | ● | NI | ○ | ● | ○ | NI | ○ | NI | NI | ● | NI | NI | NI | NI | ○ | NI | NI | NI | NI | ● | NI | NI |
| D13S227 | 13q14.3-21.1 | NI | NI | NI | ○ | ○ | NI | NI | ○ | NI | ○ | ○ | ● | ○ | NI | NI | ● | ○ | NI | ● | NI | ○ | ● | ○ | ● |
| D13S172 | 13q14.3-21.1 | ● | ○ | ○ | NI | ○ | ○ | NI | ○ | NI | ○ | ○ | NI | NI | NI | NI | NI | ○ | NI | ○ | NI | ○ | ● | NI | ● |
| D13S270 | 13q14.3-21.1 | ○ | NI | ● | NI | ○ | NI | ○ | ○ | ○ | NI | ○ | ● | NI | ○ | ○ | NI | NI | NI | ● | NI | NI | ○ | ○ | NI |
| D16S419 | 16q12.1 | ○ | ○ | NI | ○ | NI | ● | NI | NI | ○ | NI | NI | NI | NI | NI | NI | ● | ○ | NI | ○ | NI | ○ | NI | NI | ○ |
| D16S503 | 16q21 | NI | ○ | ● | ○ | NI | NI | NI | NI | ○ | NI | NI | NI | NI | ○ | ● | NI | NI | ● | NI | NI | NI | NI | ○ | ○ |
| D16S512 | 16q22.1 | ○ | NI | ● | NI | NI | NI | NI | ○ | NI | ● | ○ | ● | NI | ○ | ● | ● | NI | ● | NI | ○ | NI | NI | ○ | ○ |
| D16S515 | 16q | ○ | ○ | ● | NI | ● | ● | ○ | ○ | ○ | ● | NI | ○ | ○ | ○ | ● | ● | NI | NI | NI | ○ | ○ | ○ | NI | NI |
| D16S516 | 16q24.1 | NI | NI | ● | ○ | NI | NI | NI | ○ | ○ | NI | NI | ● | ● | NI | NI | NI | NI | NI | NI | ○ | ○ | ○ | NI | NI |
| D16S402 | 16q24.2 | ○ | ○ | NI | ○ | ● | NI | ○ | ○ | ○ | ● | ○ | ● | ○ | NI | ● | ○ | ● | ● | ○ | NI | NI | NI | NI | NI |
| D17S1176 | 17p13.1 | NI | ● | ● | ● | NI | NI | NI | ○ | ○ | NI | NI | ● | NI | ● | ● | ● | ○ | ● | ○ | ○ | ○ | ○ | ● | ● |
| TP53 | 17p13.1 | ● | ● | ● | NI | NI | ● | NI | ○ | ○ | ○ | ● | NI | NI | ● | NI | NI | NI | NI | NI | NI | NI | NI | ● | ○ |
| VNTR | 17p | ○ | ● | ○ | ○ | ○ | NI | ○ | ○ | ○ | NI | ○ | ● | ● | ● | ● | NI | NI | ○ | NI | ○ | NI | ○ | ● | ● |
| D17S520 | 17p12 | ● | ● | ● | ○ | ○ | ● | ○ | ○ | ● | ○ | ● | ○ | NI | NI | NI | ○ | ● | NI | NI | ● | NI | NI | ○ | ● |
| FAL[a] (%) | | 50 | 31 | 71 | 25 | 40 | 73 | 29 | 20 | 20 | 25 | 20 | 56 | 54 | 43 | 91 | 50 | 33 | 42 | 17 | 10 | 0 | 23 | 23 | 38 |

● Polymorphic, loss of heterozygosity

○ Polymorphic, no loss

NI Not informative

[a] Fractional allelic loss = Loss of heterozygosity of polymorphic markers / number of polymorphic markers

**Figure 3** Mutation of p53 gene and loss of heterozygosity (LOH) of chromosomes 1p, 4q, 13q, 16q and 17p. Loss of chromosome 4q is present in 75% of hepatocellular carcinomas with a p53 gene mutation

© Cancer Research Campaign 1999

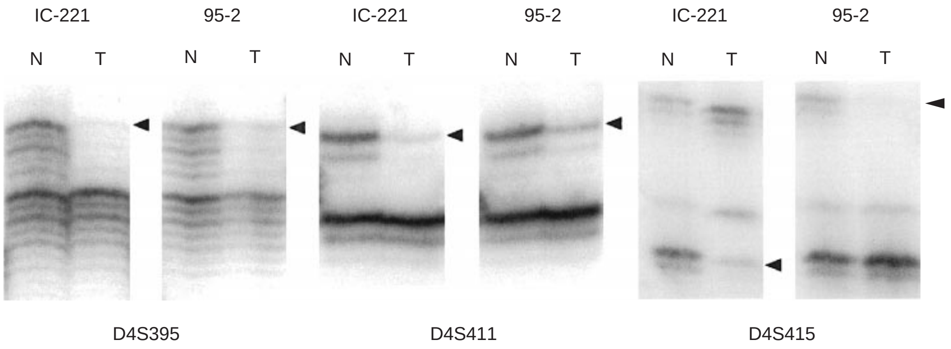

**Figure 4** Loss of heterozygosity of chromosome 4q of two tumours (IC-221 and 95-2), using three different microsatellite markers (D4S395, D4S411 and D4S415). N = normal, T = tumour. Arrowheads indicates loss of a band in a tumour lane present in the normal lane

**Table 2** LOH of microsatellite markers in HCC

| Microsatellite marker | Chromosomal location | LOH/ informative (%) | Informative/ total (%) |
|---|---|---|---|
| D1S160 | 1p36.2 | 44 (4/9) | 38 (9/24) |
| D1S170 | 1p36.2 | 40 (4/10) | 42 (10/24) |
| D1S186 | 1p32.2 | 50 (6/12) | 50 (12/24) |
| AMY2B | 1p21 | 0 (0/14) | 58 (14/24) |
| D4S395 | 4q12–13 | 53 (6/17) | 71 (17/24) |
| D4S411 | 4q23–25 | 47 (7/15) | 63 (15/24) |
| D4S427 | 4q26–28 | 53 (8/15) | 63 (15/24) |
| D4S422 | 4q28 | 36 (4/11) | 46 (11/24) |
| D4S415 | 4q32 | 57 (8/14) | 58 (14/24) |
| D13S260 | 13q12.3 | 14 (2/14) | 58 (14/24) |
| D13S126 | 13q14.1–14.3 | 44 (4/9) | 28 (9/24) |
| D13S172 | 13q14.3–21.1 | 30 (3/10) | 42 (10/24) |
| D13S227 | 13q14.3–21.1 | 56 (5/9) | 38 (9/24) |
| D13S270 | 13q14.3–21.1 | 30 (3/10) | 42 (10/24) |
| D16S419 | 16q12.1 | 20 (2/10) | 42 (10/24) |
| D16S503 | 16q21 | 33 (3/9) | 38 (9/24) |
| D16S512 | 16q22.1 | 50 (6/12) | 50 (12/24) |
| D16S515 | 16 q | 35 (6/17) | 71 (17/24) |
| D16S516 | 16q24.1 | 33 (3/9) | 39 (9/24) |
| D16S402 | 16q24.2 | 38 (6/16) | 67 (16/24) |
| D17S1176 | 17p13.1 | 59 (10/17) | 71 (17/24) |
| TP53 | 17p13.1 | 64 (7/11) | 46 (11/24) |
| VNTR | 17 p | 39 (7/18) | 75 (18/24) |
| D17S520 | 17p12 | 53 (9/17) | 71 (17/24) |

### DISCUSSION

We studied HCCs from Qidong and Shanghai in the People's Republic of China, regions with a high and an intermediate exposure to aflatoxin respectively. All patients had prior exposure to HBV as demonstrated by amplification of HBsAg and HbxAg, by PCR from all cases, and immunohistochemical demonstration of HBsAg in non-neoplastic liver in 79% of patient samples. Histopathological evidence of chronic hepatitis was present in all cases and cirrhosis in 71% of cases. HCV was not involved in the pathogenesis of HCC in our cases, as judged by negative immunohistochemistry in all cases. There was also increased frequency of GSTM1 null phenotype in these patients, corroborating a previous report of an increased incidence of HCCs in patients with a null phenotype, but no statistically significant association was found for previously characterized mutation in epoxide hydrolase (McGlynn et al, 1995). These findings confirm previous observations that HBV infection, aflatoxin exposure, and GSTM1 are key players in the aetiology of HCC in this area of the world.

Alteration of p53 was present in 95% of HCCs in our study as demonstrated by sequencing in 50% of HCCs, positive immunohistochemistry in 54% and LOH of 17p in 70%. A similar frequency of 17p loss has been reported previously (Yumoto et al, 1995). A mis-sense mutation with replacement of arginine by serine at codon 249 has been reported previously in HCCs from Qidong, Shanghai and other geographical areas where aflatoxin and HBV are present (Bressac et al, 1991; Hsu et al, 1991; Ozturk et al, 1991; Coursaget et al, 1993). LOH of 17 p without identified p53 gene mutation or positive p53 immunohistochemistry was present in 29% of HCCs in our study. HCCs developing in chronic HBV infection with functional inactivation of wild-type p53 by binding to HBxAg, as demonstrated in primary HCCs and in a transgenic mouse model (Henkler et al, 1995; Ueda et al, 1995; Greenblatt et al, 1997), could explain some of our cases.

© Cancer Research Campaign 1999

No HCC in our study had microsatellite instability (allelic shifts in more than 40% of microsatellite markers). This corroborates a previous study which reported allelic shifts in 0 to 14% (mean of 1%) of microsatellite markers in 100 HCCs using 295 microsatellite markers (Boige et al, 1997).

LOH of chromosome 1p was present in 46% of HCCs in our study. Trisomy chromosome 1, and deletions and translocations of the short arm of chromosome 1 (1p) are frequent in HCCs by cytogenetic analysis (Simon et al, 1991; Chen et al, 1996), in situ hybridization (Nasarek et al, 1995), RFLP (Simon et al, 1991) and microsatellite analysis (Yeh et al, 1994; Kuroki et al, 1995*a*, 1995*b*). LOH of chromosome 1p is an early event in hepatocarcinogenesis as evidenced by allelic loss in early or well-differentiated HCCs without loss of other chromosomal arms frequently lost in HCCs (Kuroki et al, 1995*b*). We observed loss of the telomeric portion of 1p, which is consistent with the previous study (Yeh et al, 1994). Allelic loss of 1p is also reported in other malignant tumours, such as neuroblastoma (Weith et al, 1989), colon cancer (Leister et al, 1990), breast cancer (Devilee et al, 1991) and malignant melanoma (Dracopoli et al, 1989).

LOH of 13q was present in 38% of HCCs in our study with microsatellite markers mapped to a region between 13q12.3 and 13q21.1. This region has been previously shown to include two important loci, the retinoblastoma and BRCA2 genes, and alterations of these genes are present in a subset of HCCs (Zhang et al, 1994; Kuroki et al, 1995*a*; Yumoto et al, 1995; Katagiri et al, 1996).

LOH of 16q was present in 42% of HCCs in our study. Loss of 16q is a late event in hepatocarcinogenesis and is more frequent in HCCs of poor differentiation, of larger size, and with metastasis (Tsuda et al, 1990; Yumoto et al, 1995). Reduced expression of the E-cadherin gene is associated with LOH of 16q (Slagle et al, 1993).

LOH of 4q was present in 50% of HCCs in the present study, 42–77% of HCCs in previous studies (Buetow et al, 1989; Fujimori et al, 1991; Yumoto et al, 1995; Yeh et al, 1996; Boige et al, 1997; Marchio et al, 1997; Nagai et al, 1997) and in a human hepatoma cell line (Urano et al, 1991). This loss of chromosome 4q maps to at least two regions (4q12–21, 4q22–24 and possibly an additional telomeric location) and may represent one or more tumour suppressor genes (Yeh et al, 1996; Boige et al, 1997; Marchio et al, 1997; Nagai et al, 1997). Of note, in the present study, five of ten HCCs with loss of two or more markers had retention of an intervening marker suggesting loss of two separate loci on chromosome 4q. Loss of 4q was associated with an elevated serum α-fetoprotein in HCCs patients from Taiwan (Yeh et al, 1996). Loss of chromosome 4q has also been reported in head and neck squamous cell carcinoma (Perhouse et al, 1997), cervical carcinoma (Mitra et al, 1994; Hampton et al, 1996), bladder carcinoma (Polascik et al, 1995), oesophageal and gastric cardia adenocarcinomas (Hammoud et al, 1996; Gleeson et al, 1997) and Hodgkin's disease (Dohner et al, 1992).

Introduction of a normal human chromosome 4 into immortal cell lines results in loss of proliferation and reversal of the immortal phenotype (Ning et al, 1991). A gene in the human chromosome 4q25–34 can complement the inability of a Chinese hamster mutant cell line to inhibit DNA synthesis, without altering cell survival and chromosomal stability after irradiation (Verhaegh et al, 1995). A potential candidate for a tumour suppressor gene is PTPN13, a Fas-associated protein tyrosine phosphatase which binds to a negative regulatory domain in FAS protein and inhibits FAS-induced apoptosis (Inazawa et al, 1996). This gene is located at 4q21.3 which is adjacent to marker D4S411 used in this study. Other potential candidates are caspase 3 (CPP-32/apopain) and caspase 6 (Mch2), mammalian homologues of *Ced-3* gene, and are responsible for cleavage and inactivation of key homeostatic protein during apoptosis (Nasir et al, 1997). The caspase 3 gene is located at 4q34 which is telomeric to marker D4S415, and the caspase 6 gene is located at 4q24–25. The loss of a gene(s) involved in apoptosis may complement the effects of p53 mutation, especially aflatoxin-associated p53 mutation as has been demonstrated in vitro (Ponchel et al, 1994; Dumenco et al, 1995). Transfection of a mutant p53 gene with serine at the codon 249 in a human p53-deficient hepatoma cell line induces increased in vitro survival and mitotic activity but has no effect on tumorigenicity in nude mice or apoptosis (Ponchel et al, 1994). Similarly, transfection of a mutant murine p53 gene coding for serine at the codon 246 (equivalent to human codon 249) in a murine hepatocyte cell line resulted in an increase in colony number and size, and improved growth in serum free conditions but did not cause transformation (Dumenco et al, 1995).

Fractional allelic loss (FAL) in our study ranged from 0% to 91% with a median of 32%. Two previous studies, using 195 and 275 microsatellite markers on all the chromosomes, have demonstrated less frequent FAL in HCCs with a range of 0–40% and 0–42%, respectively, and mean of 15% and 12% (Boige et al, 1997; Nagai et al, 1997). The higher frequency of FAL in our study reflects evaluation of markers on chromosomal arms which are preferentially lost in hepatocarcinogenesis.

The evaluation of multiple alterations in our study permitted us to identify interactions among environmental and genetic events. We found that LOH of 4 q was present in 75% of HCCs with a p53 gene mutation, but in only 25% of HCCs without a p53 gene mutation ($P = 0.01$). The association of 4q loss with p53 mutation is not explained by generalized allelic loses after p53 inactivation because 1p, 13q and 16q losses were not increased in HCC with p53 mutation. Additional studies are needed to see if this concerted loss of 4q in HCCs from high incidence areas with a p53 mutation is also present in HCCs from low incidence areas.

### REFERENCES

Aguilar F, Hussain SP and Cerutti P (1993) Aflatoxin $B_1$ induces the transversion of G-T in codon 249 of the tumor suppressor gene in human hepatocytes. *Proc Natl Acad Sci USA* **90**: 8586–8590

Bankfalvi A, Navabi H, Bier B, Bocker W, Jasani B and Schmid KW (1994) Wet autoclave pretreatment for antigen retrieval in diagnostic immunohistochemistry. *J Pathol* **174**: 223–228

Becker SA, Zhou Y-Z and Slagle BL (1996) Frequent loss of chromosome 8p in hepatitis B virus-positive hepatocellular carcinomas from China. *Cancer Res* **56**: 5092–5097

Boige V, Laurent-Puig P, Fouchet P, Flejou JF, Monges G, Bedossa P, Bioulac-Sage P, Capron F, Schmitz A, Olschwang S and Thomas G (1997) Concerted nonsyntenic allelic losses in hyperploid hepatocellular carcinoma as determined by a high-resolution allelotype. *Cancer Res* **57**: 1986–1990

Bourdon J-C, D'Errico A, Paterlini P, Grigioni W, May E and Debuire B (1995) p53 protein accumulation in European hepatocellular carcinoma is not always dependent on p53 gene mutation. *Gastroenterology* **108**: 1176–1182

Bressac B, Kew M, Wands J and Ozturk M (1991) Selective G to T mutations of p53 gene in hepatocellular carcinoma from southern Africa. *Nature (Lond)* **350**: 429–431

Brockmoller J, Kerb R, Drakoulis N, Nitz M and Roots I (1993) Genotype and phenotype of glutathione S-transferase class μ isoenzymes μ and ψ in lung cancer patients and control. *Cancer Res* **53**: 1004–1011

Buetow KH, Murray JC, Israel JL, London WT, Smith M, Kew M, Blanquet V, Brechot C, Redeker A and Govindarajah S (1989) Loss of heterozygosity suggests tumor suppressor gene responsible for primary hepatocellular carcinoma. *Proc Natl Acad Sci USA* **86**: 8852–8856

 © Cancer Research Campaign 1999

Chen H-L, Chen Y-C and Chen D-S (1996) Chromosome 1p aberrations are frequent in human primary hepatocellular carcinoma. *Cancer Genet Cytogenet* **86**: 102–106

Comstock KE, Sanderson BJS, Claflin G and Henner WD (1990) GST1 gene deletion determined by polymerase chain reaction. *Nucleic Acids Res* **18**: 3670

Coursaget P, Depril N, Chabaud M, Nandi R, Mayelo V, LeCann P and Yvonnet B (1993) High prevalence of mutations at codon 249 of the p53 gene in hepatocellular carcinomas from Senegal. *Br J Cancer* **67**: 1395–1397

De Souza AT, Hankins GR, Washington MK, Orton TC and Jirtle RL (1995) M6P/IGF2R gene is mutated in human hepatocellular carcinomas with loss of heterozygosity. *Nature Genet* **11**: 447–449

Devilee P, Vliet M, Bardoel A, Kievits T, Kuipers-Dijkshoon N, Pearson PL and Cornelisse CJ (1991) Frequent somatic imbalance of marker alleles for chromosome 1 in human primary breast carcinoma. *Cancer Res* **51**: 1020–1025

Dohner H, Bloomfield CD, Frizzera G, Frestedt J and Arthur DC (1992) Recurring chromosome abnormalities in Hodgkin's disease. *Genes Chromosom Cancer* **5**: 392–398

Dracopoli NC, Harnett P, Bale SJ, Stanger BZ, Tucker MA, Housman DE and Kefford RF (1989) Loss of alleles from the distal short arm of chromosome 1 occurs late in melanoma tumor progression. *Proc Natl Acad Sci USA* **88**: 4614–4618

Dumenco L, Oguey D, Wu J, Messier N and Fausto N (1995) Introduction of a murine p53 mutation corresponding to human codon 249 into a murine hepatocyte cell line results in growth advantage, but not in transformation. *Hepatology* **22**: 1279–1288

Emi M, Fujiwara Y, Nakajima T, Tsuchiya E, Tsuda H, Hirohashi S, Maeda Y, Tsuruta K, Miyaki M and Nakamura Y (1992) Frequent loss of heterozygosity for loci on chromosome 8 p in hepatocellular carcinoma, colorectal cancer, and lung cancer. *Cancer Res* **52**: 5368–5372

Fujimori M, Tokino T, Hino O, Kitagawa T, Imamura T, Okamoto E, Mitsunobu M, Ishikawa T, Nakagama H, Harada H, Yagura M, Matsubara K and Nakamura Y (1991) Allelotype study of primary hepatocellular carcinoma. *Cancer Res* **51**: 89–93

Gleeson CM, Sloan JM, McGuigan JA, Ritchie AJ, Weber JL and Russell SHE (1997) Allelotype analysis of adenocarcinoma of the gastric cardia. *Br J Cancer* **76**: 1455–1465

Greenblatt MS, Feitelson MA, Zhu M, Bennett WP, Welsh JA, Borkowski A and Harris CC (1997) Integrity of p53 in hepatitis B X antigen-positive and -negative hepatocellular carcinoma. *Cancer Res* **57**: 426–432

Groopman JD, Wang J-S and Scholl P (1996) Molecular biomarkers for aflatoxins: from adducts to gene mutations to human liver cancer. *Can J Physiol Pharmacol* **74**: 203–209

Hammoud ZT, Kaleem Z, Cooper JD, Sundaresan S, Patterson GA and Goodfellow PJ (1996) Alleotype analysis of esophageal adenocarcinomas: evidence for the involvement of sequence on the long arm of chromosome 4. *Cancer Res* **56**: 4499–4502

Hampton GM, Larson AA, Baergen RN, Sommers RL, Kern S and Cavenee WK (1996) Simultaneous assessment of loss of heterozygosity at multiple microsatellite loci using semi-automated fluorescence-based detection: subregional mapping of chromosome 4 in cervical carcinoma. *Proc Natl Acad Sci USA* **93**: 6704–6709

Hayashi H, Sugio K, Matsumata T, Adachi E, Takenaka K and Sugimachi K (1995) The clinical significance of p53 gene mutation in hepatocelluar carcinomas from Japan. *Hepatology* **22**: 1702–1707

Henkler F, Waseem N, Golding MHC, Alison MR and Koshy R (1995) Mutant p53 but not hepatitis B virus X protein is present in hepatitis B virus related human hepatocellular carcinoma. *Cancer Res* **55**: 6084–6091

Hsu H-C, Peng S-Y, Lai P-L, Sheu J-C, Chen D-S, Lin L-I, Slagle BL and Butel JS (1994) Allelotype and loss of heterozygosity of p53 in primary and recurrent hepatocellular carcinomas. *Cancer* **73**: 42–47

Hsu IC, Metcalf RA, Sun T, Welsh JA, Wang NJ and Harris CC (1991) Mutational hotspot in the p53 gene in human hepatocellular carcinomas. *Nature (Lond)* **350**: 427–428

Hsu IC, Tokiwa T, Bennett W, Metcalf RA, Welsh JA, Sun T and Harris CC (1993) p53 gene mutation and integrated hepatitis B viral DNA sequences in human cancer cell lines. *Carcinogenesis (Lond)* **14**: 987–992

Inazawa J, Ariyama T, Abe T, Druck T, Ohta M, Huebner K, Yanagisawa J, Reed JC and Sato T (1996) PTPN13, a Fas-associated protein tyrosine phosphatase, is located on the long arm of chromosome 4 at band q21.3. *Genomics* **31**: 240–242

Katagiri T, Nakamura Y and Miki Y (1996) Mutations in the BRCA2 gene in hepatocellular carcinomas. *Cancer Res* **56**: 4575–4577

Kubicka S, Trautwein C, Schrem H, Tillmann H and Manns M (1995) Low incidence of p53 mutations in European hepatocellular carcinoma with heterogeneous mutation as a rare event. *J Hepatol* **23**: 412–419

Kuroki T, Fujiwara Y, Nakamori S, Imaoka S, Kanematsu T and Nakamura Y (1995*a*) Evidence for the presence of two tumour-suppressor genes for hepatocellular carcinoma on chromosome 13 q. *Br J Cancer* **72**: 383–385

Kuroki T, Fujiwara Y, Tsuchiya E, Nakamori S, Imaoka S, Kanematsu T and Nakamura Y (1995*b*) Accumulation of genetic changes during development and progression of hepatocellular carcinoma: loss of heterozygosity on chromosome arm 1p occurs at an early stage of hepatocarcinogenesis. *Genes Chromosom Cancer* **13**: 163–167

Leister I, Weith A, Bruderlein S, Cziepluch C, Kangwanpong D, Schlag P and Schwab M (1990) Human colorectal cancer: high frequency of deletions at chromosome 1p35. *Cancer Res* **50**: 7232–7235

Leon M and Kew MC (1996) Loss of heterozygosity in chromosome 4q12–q13 in hepatocellular carcinoma in Southern African blacks. *Anticancer Res* **16**: 349–352

Mace K, Aguilar F, Wang J-S, Vautravers P, Gomez-Lechon M, Gonzalez FJ, Groopman J, Harris CC and Pfeifer AMA (1991) Aflatoxin $B_1$-induced DNA adduct formation and p53 mutations in CYP450-expressing human liver cell lines. *Carcinogenesis (Lond)* **7**: 1291–1297

Marchio A, Meddeb M, Pineau P, Danglot G, Tiollais P, Bernheim A and Dejean A (1997) Recurrent chromosomal abnormalities in hepatocellular carcinoma detected by comparative genomic hybridization. *Genes Chromosom Cancer* **18**: 59–65

McGlynn KA, Rosvold EA, Lustbader ED, Hu Y, Clapper ML, Zhou T, Wild CP, Xia X-L, Baffoe-Bonnie A, Ofori-Adjei D, Chen G-C, London WT, Shen F-M and Buetow KH (1995) Susceptibility to hepatocellular carcinoma is associated with genetic variation in the enzymatic detoxification of aflatoxin $B_1$. *Proc Natl Acad Sci USA* **92**: 2384–2387

Mitra AB, Murty VVVS, Li RG, Pratap M, Luthra UK and Chaganti RSK (1994) Allelotype analysis of cervical carcinoma. *Cancer Res* **54**: 4481–4487

Nagai H, Pineau P, Tiollais P, Buendia MA and Dejean A (1997) Comprehensive allelotyping of human hepatocellular carcinoma. *Oncogene* **14**: 2927–2933

Nasarek A, Werner M, Nolte M, Klempnauer J and Georgii A (1995) Trisomy 1 and 8 occur frequently in hepatocellular carcinoma but not in liver cell adenoma and focal nodular hyperplasia. A fluorescence in situ hybridization study. *Virchows Arch* **427**: 373–378

Nasir J, Theilmann JL, Chopra V, Jones AM, Walker D, Rasper DM, Vaillancourt JP, Hewitt JE, Nicholson DW and Hayden MR (1997) Localization of the cell death genes CPP32 and Mch-2 to human chromosome 4q. *Mammalian Genome* **8**: 56–59

Ning Y, Weber JL, Killary AM, Ledbetter DH, Smith JR and Pereira-Smith OM (1991) Genetic analysis of indefinite division in human cells: evidence for a cell senescence-related gene(s) on human chromosome 4. *Proc Natl Acad Sci USA* **88**: 5635–5639

Nose H, Imazeki F, Ohto M and Omata M (1993) p53 gene mutations and 17p allelic deletions in hepatocellular carcinoma from Japan. *Cancer* **72**: 355–360

Ozturk M, Bressac B, Puisieux A, Kew M, Volkmann M, Bozcall S, Bella Mura J, de la Monte S, Carlson R, Blum H, Wands J, Takahashi H, von Weizsäcker F, Galun E, Siddhartha K, Carr BI, Schröder CH, Erken E, Varinli S, Rustgi VK, Prat J, Toda G, Koch HK, Liang XH, Tang Z-Y, Shouval D, Lee H-S, Vyas GN and Sarosi I (1991) p53 mutation in hepatocellular carcinoma after aflatoxin exposure. Lancet **338**: 1356–1359.

Pershouse MA, El-Naggar AK, Hurr K, Lin H, Yung WKA and Steck PA (1997) Deletion mapping of chromosome 4 in head and neck squamous cell carcinoma. *Oncogene* **14**: 369–373

Polascik TJ, Cairns P, Chang WYH, Schoenberg MP and Sidransky D (1995) Distinct regions of allelic loss on chromosome 4 in human primary bladder carcinoma. *Cancer Res* **55**: 5396–5399

Ponchel F, Puisieux A, Tabone E, Michot JP, Froschl G, Morel AP, Frebourg T, Fontaniere B, Oberhammer F and Ozturk M (1994) Hepatocarcinoma-specific mutant p53-249ser induces mitotic activity but has no effect on transforming growth factor β1-mediated apoptosis. *Cancer Res* **54**: 2064–2068

Qian G-S, Ross RK, Yu, MC, Yuan, J-M, Gao Y-T, Henderson BE, Wogan GN and Groopman JD (1994) A follow-up study of urinary markers of aflatoxin exposure and liver cancer risk in Shanghai, People's Republic of China. *Cancer Epidemiol Biomarkers Prev* **3**: 3–10

Rashid A and Hamilton SR (1997) Genetic alterations in sporadic and Crohn's-associated adenocarcinomas of the small intestine. *Gastroenterology* **113**: 127–135

Redston MS, Caldas C, Seymour AB, Hruban RH, da Costa L, Yeo CJ and Kern SE (1994) p53 mutations in pancreatic carcinoma and evidence of common involvement of homocopolymer tracts in DNA microdeletions. *Cancer Res* **54**: 3025–3033

Shi CY, Phang TW, Lin Y, Wee A, Li B, Lee HP and Ong CN (1995) Codon 249 mutation of the p53 gene is a rare event in hepatocellular carcinomas from ethnic Chinese in Singapore. *Br J Cancer* **72**: 146–149

*© Cancer Research Campaign 1999* 

Simon D, Knowles BB and Weith A (1991) Abnormalities of chromosome 1 and loss of heterozygosity on 1p in primary hepatomas. *Oncogene* **6**: 765–770

Slagle BL, Zhou Y-Z, Birchmeier W and Scorsone KA (1993) Deletion of the E-cadherin gene in hepatitis B virus-positive Chinese hepatocellular carcinomas. *Hepatology* **18**: 757–762

Takahashi K, Kudo J, Ishibashi H, Hirata Y and Niho Y (1993) Frequent loss of heterozygosity on chromosome 22 in hepatocellular carcinoma. *Hepatology* **17**: 794–799

Tsuda H, Zhang W, Shimosato Y, Yokota J, Terada M, Sugimura T, Miyamura T and Hirohashi S (1990) Allele loss on chromosome 16 associated with progression of human hepatocellular carcinoma. *Proc Natl Acad Sci USA* **87**: 6791–6794

Ueda H, Ullrich SJ, Gangemi JD, Kappel CA, Ngo L, Feitelson MA and Jay G (1995) Functional inactivation but not structural mutation of p53 causes liver cancer. *Nature Genet* **9**: 41–47

Urano Y, Watanabe K, Lin CC, Hino O and Tamaoki T (1991) Interstitial chromosomal deletion within 4q11–q13 in a human hepatoma cell line. *Cancer Res* **51**: 1460–1464

Verhaegh GWCT, Jongmans W, Jaspers NGJ, Natarajan AT, Oshimura M, Lohman PHM and Zdzienicka MZ (1995) A gene that regulates DNA replication in response to DNA damage is located on human chromosome 4q. *Am J Hum Genet* **57**: 1095–1103

Walker GJ, Hayward NK, Falvey S and Cooksley WGE (1991) Loss of somatic heterozygosity in hepatocellular carcinoma. *Cancer Res* **51**: 4367–4370

Wang HP and Rogler CE (1988) Deletions in human chromosome arms 11p and 13q in primary hepatocellular carcinomas. *Cytogenet Cell Genet* **48**: 72–78

Weith A, Martinsson T, Cziepluch C, Bruderlein S, Amler LC, Berthold F and Schwab M (1989) Neuroblastoma consensus deletion maps to 1p36.1-2. *Genes Chromosom Cancer* **1**: 159–166

Yeh S-H, Chen P-J, Chen H-L, Lai M-Y, Wang C-C and Chen D-S (1994) Frequent genetic alterations at the distal region of chromosome 1p in human hepatocellular carcinomas. *Cancer Res* **54**: 4188–4192

Yeh S-H, Chen P-J, Lai M-Y and Chen D-S (1996) Allelic loss on chromosomes 4q and 16q in hepatocellular carcinoma: association with elevated α-fetoprotein production. *Gastroenterology* **110**: 184–192

Yu M-W and Chen C-J (1994) Hepatitis B and C viruses in the development of hepatocellular carcinoma. *Crit Rev Oncol Hematol* **17**: 71–91

Yumoto Y, Hanafusa T, Hada H, Morita T, Ooguchi S, Shinji N, Mitani T, Hamaya K, Koide N and Tsuji T (1995) Loss of heterozygosity and analysis of mutation of p53 in hepatocellular carcinoma. *J Gastroenterol Hepatol* **10**: 179–185

Zhang W, Hirohashi S, Tsuda H, Shimosato Y, Yokota J, Terada M and Sugimura T (1990) Frequent loss of heterozygosity on chromosomes 16 and 4 in human hepatocellular carcinoma. *Jpn J Cancer Res* **81**: 108–111

Zhang X, Xu H-J, Murakami Y, Sachse R, Yashima K, Hirohashi S, Hu S-X, Benedict WF and Sekiya T (1994) Deletions of chromosome 13q, mutations in retinoblastoma 1, and retinoblastoma protein state in human hepatocellular carcinoma. *Cancer Res* **54**: 4177–4182

*© Cancer Research Campaign 1999*
